# In-Vitro Activity of Polymyxin B, Rifampicin, Tigecycline Alone and in Combination against Carbapenem-Resistant *Acinetobacter baumannii* in Singapore

**DOI:** 10.1371/journal.pone.0018485

**Published:** 2011-04-21

**Authors:** Tze-Peng Lim, Thean-Yen Tan, Winnie Lee, S. Sasikala, Thuan-Tong Tan, Li-Yang Hsu, Andrea L. Kwa

**Affiliations:** 1 Department of Pharmacy, Singapore General Hospital, Singapore, Singapore; 2 Department of Microbiology, Changi General Hospital, Singapore, Singapore; 3 Singapore General Hospital, Singapore, Singapore; 4 Division of Infectious Diseases, Department of Medicine, Yong Loo Lin School of Medicine, National University of Singapore, Singapore, Singapore; East Carolina University School of Medicine, United States of America

## Abstract

**Objective:**

Carbapenem-resistant *Acinetobacter baumannii* (CR-AB) is an emerging cause of nosocomial infections worldwide. Combination therapy may be the only viable option until new antibiotics become available. The objective of this study is to identify potential antimicrobial combinations against CR-AB isolated from our local hospitals.

**Methods:**

AB isolates from all public hospitals in Singapore were systematically collected between 2006 and 2007. MICs were determined according to CLSI guidelines. All CR-AB isolates were genotyped using a PCR-based method. Clonal relationship was elucidated. Time-kill studies (TKS) were conducted with polymyxin B, rifampicin and tigecycline alone and in combination using clinically relevant (achievable) unbound concentrations.

**Results:**

31 CR AB isolates were identified. They are multidrug-resistant, but are susceptible to polymyxin B. From clonal typing, 8 clonal groups were identified and 11 isolates exhibited clonal diversity. In single TKS, polymyxin B, rifampicin and tigecycline alone did not exhibit bactericidal activity at 24 hours. In combination TKS, polymyxin plus rifampicin, polymyxin B plus tigecycline and tigecycline plus rifampicin exhibited bactericidal killing in 13/31, 9/31 and 7/31 isolates respectively at 24 hours. Within a clonal group, there may be no consensus with the types of antibiotics combinations that could still kill effectively.

**Conclusion:**

Monotherapy with polymyxin B may not be adequate against polymyxin B susceptible AB isolates. These findings demonstrate that in-vitro synergy of antibiotic combinations in CR AB may be strain dependant. It may guide us in choosing a pre-emptive therapy for CR AB infections and warrants further investigations.

## Introduction

*Acinetobacter baumannii* is a successful pathogen that has evoked scientific and public interest worldwide.[1] It is increasingly multidrug-resistant due to its wide repertoire of antimicrobial resistance mechanisms and its innate ability to acquire new resistance determinants.[2] As a frequently occurring pathogen associated with serious nosocomial infections, *A. baumannii* had been shown to be associated with unfavourable clinical outcomes.[3], [4]

The antibiotic development pipeline is under pressure from the alarming spread of antimicrobial resistance and for all practical purposes is completely dry. Thus, for treatment of these difficult-to-treat non-fermentative organisms, we are now in the pre-antibiotic era. Carbapenems, members of a potent broad-spectrum antimicrobial class, are increasingly being used as first-line therapy in institutions where there is a high prevalence of multidrug-resistant bacterial infections. With the advent of increasing usage and poor infection control, carbapenem resistance has emerged worldwide and there has been a surge in recent reports of outbreaks involving multidrug-resistant *A. baumannii* that are also carbapenem-resistant (CR-AB).[5], [6] This phenomena has resulted in the revival of the polymyxins, which are increasingly used as the last line of defence against such difficult-to-treat infections.

Inevitably, growing reports of polymyxin heteroresistance in CR-AB and even pan drug-*resistant A. baumannii* (PDR-AB) have come to light.[7], [8] These evidences had suggested against the use of polymyxins as monotherapy. Tigecycline is a new glycylcycline that showed good in-vitro activity against multidrug-resistant *A. baumannii* isolates. However, its clinical utility has yet to be demonstrated alone or in combination with other antibiotics.[9] Other than stringent infection control measures, combination therapy may be our only current remaining viable therapeutic option in treating infections caused by such bacteria. Therefore, the objective of this study was to identify potential bactericidal antimicrobial combinations against CR-AB in Singapore.

## Materials and Methods

### Antimicrobial agents

Polymyxin B and rifampicin were obtained from Sigma-Aldrich (St. Louis, MO). Tigecycline was obtained from Wyeth Pharmaceuticals (Pearl River, NY). For polymyxin B, a stock solution in sterile water was prepared, aliquoted, and stored at −70°C. Tigecycline in solution was freshly prepared before each experiment. On the other hand, rifampicin was dissolved in dimethyl sulfoxide and was then serially diluted in sterile water to the desired final drug concentration. The final dimethyl sulfoxide concentration had no effect on *A. baumannii* growth. Prior to each susceptibility test, an aliquot of the drug was thawed and diluted to the desired concentrations with Ca-MHB.

### Microorganisms and susceptibility testing

*Acinetobacter baumannii* isolates from the urinary tract, blood and respiratory tract were collected from five geographically separate hospitals over a two-year period (2006–2007) by Network for Antimicrobial Resistance Surveillance (Singapore). These CR-AB isolates were previously described harbouring the *bla*OXA-23-like and *bla*OXA-51-like carbapenemase genes, with the ISAba1 upstream of the *bla*OXA-23 gene (results not shown).[10] Genus identity was initially determined using conventional biochemical methods and ID-GN cards (Vitek 2, bioMérieux, France) and confirmed by PCR-based method.[11] Minimum inhibitory concentrations (MIC) to ampicillin/sulbactam, ciprofloxacin, gentamicin, imipenem, meropenem, aztreonam, piperacillin/tazobactam, polymyxin B, tigecycline, ceftazidime, amikacin and cefepime were obtained by microbroth dilution. MICs to rifampicin were obtained by a modified broth macrodilution method as described by the CLSI.[12] CR-AB were defined as isolates resistant to all tested antibiotics classes except polymyxins.[13] The bacteria were stored at −70°C in Protect® (Key Scientific Products, Stamford, TX, USA) storage vials. Fresh isolates were sub-cultured twice on 5% blood agar plates (Biomedia-Bloxwich, Malaysia) for 24 h at 35°C prior to each experiment.

### Clonal Relationship Analysis

All study isolates were genotyped using a PCR-based method.[14] Digital images of the DNA fingerprints were processed using Gene Profiler 4.05 (Scanalytics, BD Biosciences, USA) and similarity analysis, distance estimation and cluster analysis using UPGMA were performed using Treecon software.

### Time-kill studies

Time-kill studies were conducted with polymyxin B, rifampicin and tigecycline alone and in combination using clinically relevant (achievable) unbound concentrations. Hence, the simulated steady-state drug concentrations were 2 mg/L (free or unbound protein fraction) for polymyxin B, rifampicin and tigecycline, with corresponding maximum intravenous doses of at least 1 million units of polymyxin B (every 12 hours), 600 mg of rifampicin (every 12 hours) and 100 mg of tigecyline (every 12 hours).[15], [16], [17]

An overnight culture of the isolate was diluted into pre-warmed cation-adjusted Mueller Hinton II broth (Ca-MHB) (BBL, BD, USA) and incubated further at 35°C until reaching log-phase growth. The bacterial suspension was diluted with Ca-MHB according to absorbance (at 630 nm); 15 ml of the suspension was transferred to 50-ml sterile conical flasks, each containing 1 ml of a drug dilution at 16 times the target concentration. The final concentration of the bacterial suspension in each flask was approximately 10^5^ CFU/ml (ranging from 1×10^5^ CFU/ml to 5×10^5^ CFU/ml).

Flasks were incubated in a shaker water bath at 35°C. Serial samples of broth were obtained from each flask at 0 (baseline), 2, 4, 8, 12 and 24 hours after incubation. Samples were obtained in duplicate at each time-point. Extracted broth samples (0.5 ml) were first centrifuged at 10,000 x *g* for 15 minutes and then reconstituted with sterile normal saline to their original volumes in order to minimize drug carryover. The total bacterial count for each sample was quantified by depositing serial 10-fold dilutions of broth samples onto Mueller Hinton agar (MHA) plates (Biomedia, Bloxwich, Malaysia) using a spiral-plater (Interscience, St Nom La Breteche, France).

Inoculated plates were incubated in a humidified incubator (35°C) for 18 to 24 h, bacterial colonies were visually counted, and the original bacterial density from the original sample was calculated based on the dilution factor. The lower limit of detection for the colony counts was 2 log_10_ CFU/ml.

### Pharmacodynamic endpoints

Bactericidal activity (primary endpoint) was defined as a ≥3 log_10_ CFU/ml decrease in the colony count from the initial inoculum at 24 hours. Synergy (secondary endpoint) was defined as a ≥2 log_10_ CFU/ml decrease in the colony count by the drug combination when compared with its most active constituent and a ≥2 log_10_ CFU/ml decrease from the initial inoculum at 24 hours while indifference was defined as a <2 log_10_ CFU/ml change at 24 hours by the combination compared with that by the most active single agent.[18]

## Results

### Susceptibility

Thirty-one CR-AB isolates were identified. All isolates were resistant to meropenem, imipenem, ampicillin/sulbactam, ciprofloxacin, gentamicin, aztreonam, piperacillin/tazobactam, ceftazidime, cefepime and amikacin. (data not shown) But they were susceptible to polymyxin B (MIC range 0.5–2 mg/L). There are no CLSI susceptibility breakpoints for rifampicin and tigecycline against *A. baumannii*. The MICs of rifampicin and tigecycline ranged from 1–≥64 mg/L and 0.5–≥32 mg/L respectively (Table 1).

**Table 1:** Susceptibilities of 31 *A. baumannii* isolates.

				Susceptibility (%)		
Antibiotics	MIC_50_ (mg/L)	MIC_90_ (mg/L)	Range (mg/L)	*R*	*I*	*S*
Polymyxin B	1	2	0.5–2	–	–	100
Rifampicin	6	≥64	1–≥64	–	–	–
Tigecycline	4	≥32	0.5–≥32	–	–	–

### Clonal Relationship Analysis

**Figure 1:**
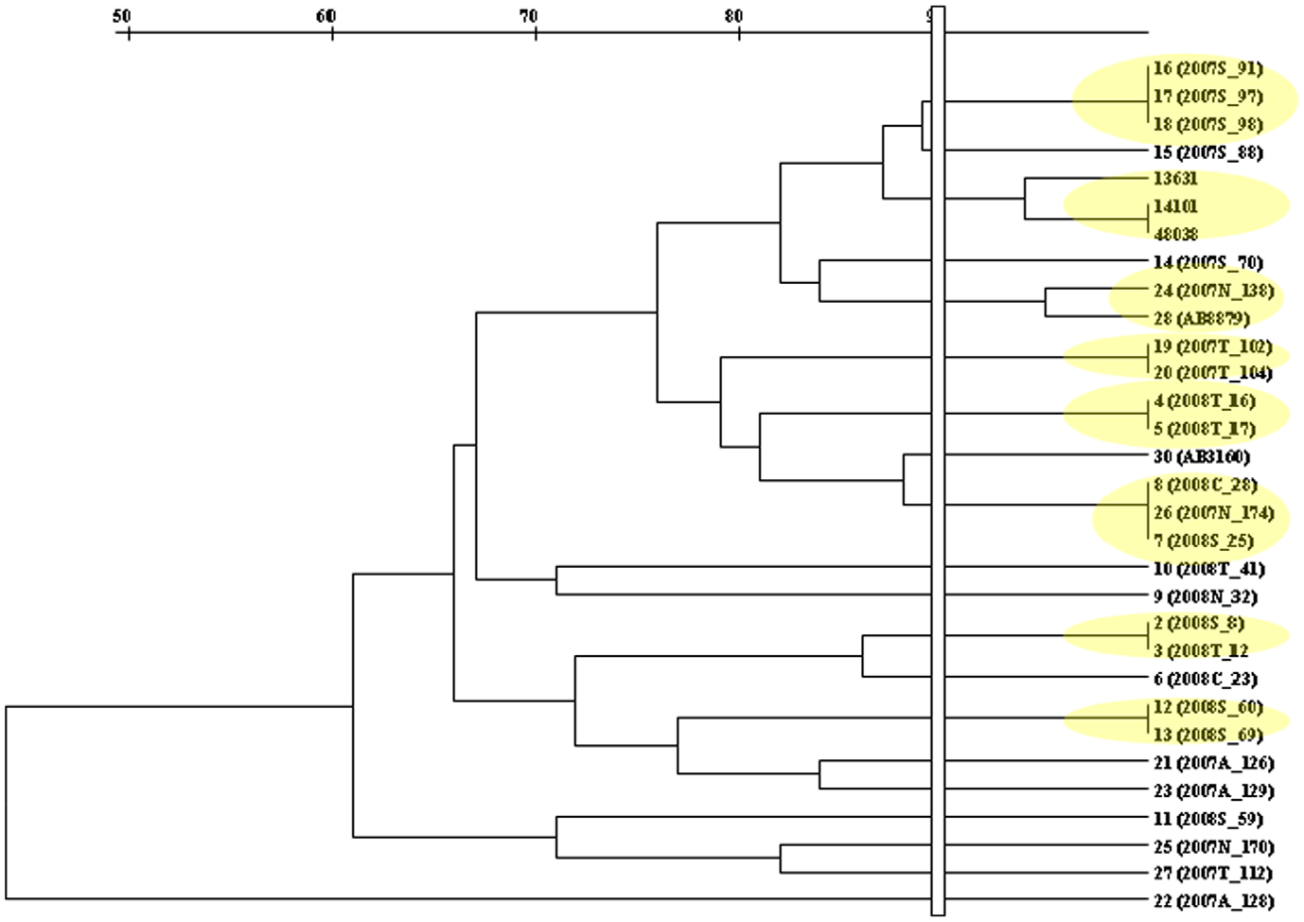
Phylogenetic Tree Diagram showing * clonal groups. A yellow oval, denote a clonal group after applying a similarity index of 90% to PCR typing results.

Applying a similarity index of 90% to PCR typing results, 8 clonal clusters were identified consisting of groups of 2 or 3 isolates for each cluster. Eleven remaining isolates showed greater clonal diversity (Figure 1).

### Time-kill studies

In time-kill studies, polymyxin B alone generally demonstrated indifferent activity in 26 out of 31 CR AB strains where there were <2 log_10_ CFU/ml decrease between the initial inocula and the 24 hr time-point in the colony counts. Five out of 31 strains showed an increase in bacteria density and a higher bacteria concentration was observed at 24 hours (∼8 log_10_ CFU/ml). Rifampicin and tigecycline alone could hardly achieve a reduction in bacterial burden respectively. The average increase in CFU/ml from baseline was greater than 2 log_10_ CFU/ml at 24 hours for all strains except for AB 17 (average 1.5 log_10_ reduction from baseline) against rifampicin and tigecycline alone and AB 32 (average 1.9 log_10_ reduction from baseline) against tigecycline alone (Table 2).

**Table 2:** 24 hour bacteria burden (log_10_ CFU/ml) after exposure to individual antibiotics.

		Tigecycline		Polymyxin B		Rifampicin	
AB strain	Baseline inoculum	Mean	SD	Mean	SD	Mean	SD
**8**	5.22	8.53	0.04	7.13	0.05	8.07	0.04
**12**	5.27	8.60	0.09	5.72	0.11	7.77	0.01
**16**	5.23	7.72	0.02	3.50	0.07	8.69	0.03
**17**	5.03	3.74	0.11	9.14	0.13	3.29	0.06
**23**	5.35	7.94	0.04	4.94	0.12	5.99	0.13
**25**	5.44	6.86	0.09	5.72	0.01	7.83	0.11
**28**	5.30	8.08	0.11	8.61	0.04	8.73	0.03
**32**	5.30	3.40	0.14	5.11	0.49	7.91	0.08
**41**	5.38	7.99	0.01	8.04	0.01	5.56	0.11
**59**	5.25	8.08	0.06	5.13	0.34	9.17	0.01
**60**	5.36	7.97	0.09	4.02	0.00	8.46	0.01
**69**	5.25	8.16	0.05	5.48	0.13	8.97	0.01
**70**	5.26	8.33	0.02	3.12	0.08	8.60	0.02
**88**	5.28	7.17	0.23	4.84	0.17	6.11	0.06
**91**	5.20	7.14	0.17	3.00	0.06	8.58	0.07
**97**	5.33	6.94	0.36	4.85	0.04	5.62	0.05
**98**	5.54	7.72	0.23	4.31	0.15	5.59	0.01
**102**	5.21	6.63	0.35	5.09	0.08	8.05	0.11
**104**	5.16	6.84	0.08	5.05	0.06	7.70	0.11
**126**	5.20	8.77	0.13	5.59	0.01	5.43	0.01
**128**	5.42	8.71	0.12	7.86	0.00	5.82	0.01
**129**	5.19	8.84	0.01	4.07	0.15	8.28	0.11
**138**	5.38	6.77	0.13	4.40	0.06	8.36	0.04
**170**	5.37	7.62	0.06	5.42	0.10	8.49	0.02
**174**	5.26	6.35	0.05	3.57	0.10	8.24	0.08
**112**	5.18	7.45	0.00	4.73	0.04	7.52	0.01
**8879**	5.01	5.25	0.04	4.23	0.05	7.56	0.02
**14101**	5.40	5.38	0.23	5.71	0.13	8.33	0.13
**3160**	5.17	8.93	0.05	4.75	0.18	8.60	0.06
**13631**	5.43	7.93	0.09	5.59	0.18	8.86	0.01
48038	5.32	8.90	0.06	4.78	0.04	8.18	0.10

For the various antibiotic combinations, polymyxin B plus rifampicin achieved the highest percentage of bactericidal activity in 13 out of 31 isolates (41.9%) and indifferent activity against the rest of the isolates; polymyxin B plus tigecycline achieved bactericidal activity in 9 out of 31 isolates (29.0%); whereas tigecycline plus rifampicin achieved the lowest percentage of bactericidal activity in 7 out of 31 isolates (22.6%) (Table 3). None of the antibiotics combinations demonstrated bactericidal activity against 14 out of 31 strains tested.

**Table 3:** 24 hour bacteria burden (log_10_ CFU/ml) after exposure to various antibiotic combinations.

	Tigecycline + Rifampicin		Polymyxin B + Rifampicin		Polymyxin B + Tigecycline	
AB strain	Mean	SD	Mean	SD	Mean	SD
**8**	6.81	0.12	**0.00**	0.00	5.11	0.08
**12**	7.19	0.01	**0.00**	0.00	**0.00**	0.00
**16**	4.70	0.00	3.56	0.11	**0.80**	1.13
**17**	**0.00**	0.00	**0.00**	0.00	**0.65**	0.92
**23**	4.95	0.11	4.85	0.17	4.82	0.06
**25**	4.73	0.00	**0.00**	0.00	5.01	0.01
**28**	5.53	0.01	3.57	0.01	4.80	0.01
**32**	5.41	0.08	**0.00**	0.00	**2.31**	0.15
**41**	4.77	0.16	4.54	0.13	5.10	0.03
**59**	8.67	0.00	4.10	0.05	3.24	0.04
**60**	6.65	0.49	5.37	0.10	**0.80**	1.13
**69**	7.64	0.06	4.10	0.11	5.08	0.08
**70**	6.84	0.11	4.66	0.08	4.09	0.01
**88**	**2.42**	0.05	**0.00**	0.00	4.90	0.05
**91**	**0.00**	0.00	**0.00**	0.00	4.65	0.09
**97**	**0.00**	0.00	**0.00**	0.00	4.25	0.06
**98**	**0.00**	0.00	**0.00**	0.00	4.84	0.04
**102**	5.35	0.04	**0.00**	0.00	4.86	0.22
**104**	4.48	0.04	4.85	0.04	4.57	0.08
**126**	**2.60**	0.00	**2.48**	0.00	**1.60**	0.42
**128**	5.49	0.01	2.69	0.06	**0.00**	0.00
**129**	5.93	0.00	4.73	0.04	**2.28**	0.18
**138**	5.33	0.01	3.86	0.06	4.15	0.28
**170**	8.29	0.01	3.79	0.10	3.66	0.00
**174**	5.59	0.01	4.65	0.07	4.63	0.18
**112**	**0.00**	0.00	**0.00**	0.00	**0.00**	0.00
**8879**	3.20	0.12	**0.00**	0.00	3.02	0.12
**14101**	4.27	0.03	5.27	0.30	7.16	0.01
**3160**	6.70	0.11	4.62	0.08	4.59	0.01
**13631**	5.91	0.10	5.44	0.06	5.94	0.06
**48038**	6.03	0.20	5.44	0.11	5.41	0.00

(Bactericidal combinations denoted in bold)

Of these 14 isolates, 8 elicited indifferent activity when combination antibiotics were used. Using synergy as the secondary endpoint to compare the activity of the antibiotic combinations, tigecycline plus rifampicin was synergistic in 3 isolates while all three antibiotic combinations showed synergistic activity against 1 isolate (AB 28). Against the remaining 2 isolates, polymyxin B alone is more effective (i.e. with the lowest bacteria burden at 24 hours compared against the baseline inocula when compared to all combination antibiotics.

Comparing the results with respect to the 8 major clonal groups of isolates, the results were in agreement for 2 clonal groups (isolates 91, 97 & 98; isolates 14101, 13631 & 48038) for all antibiotic combinations while 4 clonal groups had only 66% similarity for all combinations. The remaining 2 groups showed conflicting results where there is only 33% similarity in the results (Table 4).

**Table 4:** Clonal group analysis of the 24 hour bacteria burden (log_10_ CFU/ml) after exposure to various antibiotic combinations.

	Tigecycline + Rifampicin	Polymyxin B + Rifampicin	Polymyxin B + Tigecycline	% similarity
AB strain	Mean	Mean	Mean	
**91**	**0.00**	**0.00**	4.65	
**97**	**0.00**	**0.00**	4.25	100
**98**	**0.00**	**0.00**	4.84	
**14101**	4.27	5.27	7.16	
**13631**	5.91	5.44	5.94	100
**48038**	6.03	5.44	5.41	
**8**	6.81	**0.00**	5.11	66
**12**	7.19	**0.00**	**0.00**	
**25**	4.73	**0.00**	5.01	
**28**	5.53	3.57	4.80	66
**174**	5.59	4.65	4.63	
**60**	6.65	5.37	**0.80**	66
**69**	7.64	4.10	5.08	
**102**	5.35	**0.00**	4.86	66
**104**	4.48	4.85	4.57	
**16**	4.70	3.56	**0.80**	33
**17**	**0.00**	**0.00**	**0.65**	
**138**	5.33	3.86	4.15	33
**8879**	**3.20**	**0.00**	**3.02**	

(Bactericidal combinations denoted in bold)

## Discussion

Infections caused by carbapenem-resistant *A. baumannii* present challenges to clinicians where they are left with practically no rational choice of antimicrobial treatment. As a result, there are growing reports of such infections for which no therapeutic option exists.[13], [19] Combination therapy for the treatment of CR-AB organisms has increasingly been used although clinical trials of antibiotic combinations showing enhanced activity are extremely rare. Therefore, any antibiotic combinations that yield some success in-vitro are the only potential solution in these clinically stuck situations.

Unlike the conventional rationale when combining 2 agents is to enhance the activity of either agent through the achievement of a synergistic effect, an additional objective is to help to attain an enhanced pharmacodynamic effect that can potentially curb the emergence of further resistance.[20] As the observed CR AB phenotype can be mediated by several molecular mechanisms of resistance, an antibiotic combination that has been previously elucidated against a particular organism may not always be effective for another patient having the same infection. Within a clonal group, there may be no consensus with the types of antibiotics combinations that could still kill effectively. As AB picks up resistance determinants with great ease, one speculation could be that different antibiotic resistant mechanisms, which may be up-regulated or acquired, to a different extent during different antibiotics exposure, could possibly be found in related isolates. An additional caution is then that combination testing data could not reliably be applied to all members of a clonal group. Hence, it is also clear that efficacious antibiotic combinations against CR AB may be highly strain-specific. To the best of our knowledge, this is the first study that had objectively evaluated antibiotic combinations for thirty-one non-isogenic CR AB isolates using the time-kill method and the bactericidal activity as the pharmacological measurement of efficacy. We did not use the conventional (synergistic activity) pharmacological index as our primary measurement of efficacy as all the test isolates were resistant to all the antibiotics (i.e. the synergistic definition may no longer be applicable for CR-AB organisms in an useful manner). Although this method cannot make the results available to the clinicians in a timely manner for individual bedside decisions, it can help narrow down the possible alternative combinations to use for empiric treatment while waiting for the combination testing to be conducted for every CR AB infection.

In a similar study by Sopirala et al [21], they reported that imipenem + colistin, or imipenem + tigecycline were effective synergistic combinations for CR AB, while tigecycline + colistin combination was ineffective. In contrary, polymyxins + tigecycline was one of the promising bactericidal combination for our Singapore CR AB isolates, while polymyxins + carbapenem combination was not. This is likely due to the differences in the underlying mechanisms of resistance in our CR AB, when compared to those in Sopirala et al. Our CR-AB isolates harboured the *bla*OXA-23-like and *bla*OXA-51-like carbapenemase genes, with the ISAba1 upstream of the *bla*OXA-23 gene (results not shown), while the genetic determinants of resistance in CR AB isolates in Sopirala et al revealed class 1 integrons in all of their clones, along with OXA β-lactamases (but not extended spectrum β-lactamases *bla-PER* and 294 *bla-TEM*) that can hydrolyze carbapenems along with acetyltransferase genes *aacA4, aac(6′)-Iad*, *aacC6 and* phosphotransferase gene *aphA1*, which impart resistance to aminoglycosides. The type of OXA β-lactamases, however, was not reported. In addition, by selecting a representative clone from each of the eight clonal types for antibiotic synergy testing, Sopirala et al had assumed that the antibiotics combination, that was synergistic and effective for the representative clone, should also be effective for the rest in the same clonal family. However, our findings illustrated that within a clonal group, there may be no consensus with the types of antibiotics combinations that could still kill effectively.

Our primary objective in this study was purely to identify potential bactericidal antimicrobial combinations against CR-AB in Singapore via time-kill studies. However, while Sopirala et al aimed to determine the combination of agents which reveal *in vitro* antimicrobial synergy by two different Etest methods and broth micro-dilution checkerboard (CB) method, they also aimed to find a method that could be easily performed in clinical microbiology laboratory, and had the best correlation with time-kill studies by comparing results of two different Etest and CB methods with time-kill studies.

Overall, Sopirala's work reinforces that different antimicrobial combinations apply to different strains with different mechanisms of resistance. There are many in-vitro and animal studies that support the role of combination therapy with polymyxins against *A.baumannii*. The antibiotic combinations that had been shown to provide enhanced activity compared to any single agent include: polymyxin B or colistin plus rifampicin or azithromycin, imipenem or azithromycin plus rifampicin and the triple combination of imipenem, rifampicin and polymyxin B. In particular, polymyxins B/E in combination with rifampicin appear most promising.[22], [23], [24], [25], [26], [27], [28], [29] Our findings in addition to Sopirala's will lend clinical relevance in accordance to the geographical location and help guide the use of appropriate antibiotics combination in different parts of the world where there are different mechanisms of resistance in CR AB.

As adequate dosing of antibiotics is pertinent in extremely resistant infections, clinically achievable free or unbound concentrations from maximally possible antibiotic doses were used for all the tested antibiotics to mimic as close as possible the killing effect that takes place in vivo. Since it is the free or unbound protein fraction of a drug that is pharmacologically active, all drug exposures in the experiments were expressed as free drug concentrations at steady state.[30] As tigecycline distributes readily into the tissues and does not stay in serum, it is not indicated for the treatment of bacteraemia. Hence, we utilized an achievable average tissue concentration (that is also the CLSI breakpoint of tigecycline) in our experiments instead of the serum concentrations.[31] Based on the tigecycline breakpoint concentration, the results obtained with the polymyxin B plus tigecycline combination suggested some clinical utility in tissue infections caused by CR-AB.

Colistin heteroresistance has been reported among *Acinetobacter* isolates that are still susceptible to colistin.[8], [32] This is a worrying concern as colistin or polymyxin B is increasingly been used as monotherapy to treat micro-organisms that are reported as susceptible to colistin/polymyxin B only. There has been a recent report of isolates exhibiting heteroresistance from patients previously treated with colistin.[7] Although we did not evaluate the emergence of resistance to polymyxins in our study, our results suggested that polymyxin B did not exhibit bactericidal activity in the majority of our polymyxin B susceptible isolates. Combination therapy may be the only therapeutic option to preserve the clinical utility of the polymyxins against such resistant pathogens.

Future research should include more pharmacokinetic studies to establish more clinically relevant profiles of tigecycline and colistin/polymyxin B. This will improve the applicability of the data in in-vitro and in-vivo models to elucidate antimicrobial combinations against CR-AB that can potentially be utilized in a clinically stuck situation. In-vitro combination testing of antimicrobial combinations using clinically relevant drug concentrations instead of MIC-based concentrations is recommended to guide clinicians in determining which antimicrobial combination and at what doses are to be used as empiric therapy. Current work is in progress to reduce the time needed to determine the useful antibiotic combinations and make available the information to the clinicians in a more timely fashion.

### Conclusion

In summary, few treatment options are available for the treatment of carbapenem resistant *Acinetobacter baumannii*. The polymyxins remain the only agent that may be consistently active in-vitro against them. However, polymyxin resistant isolates are slowly emerging. Identifying novel antimicrobial combinations with proven in-vitro activity should encompass local susceptibility patterns as well as molecular mechanisms of resistance to provide greater efficacy and reduce the emergence of resistance.
